# The Intertwined Evolution and Development of Sutures and Cranial Morphology

**DOI:** 10.3389/fcell.2021.653579

**Published:** 2021-03-26

**Authors:** Heather E. White, Anjali Goswami, Abigail S. Tucker

**Affiliations:** ^1^Department of Life Sciences, Natural History Museum, London, United Kingdom; ^2^Centre for Craniofacial and Regenerative Biology, King’s College London, London, United Kingdom; ^3^Division of Biosciences, University College London, London, United Kingdom

**Keywords:** suture, morphology, development, craniofacial, evolution, mammal, skull

## Abstract

Phenotypic variation across mammals is extensive and reflects their ecological diversification into a remarkable range of habitats on every continent and in every ocean. The skull performs many functions to enable each species to thrive within its unique ecological niche, from prey acquisition, feeding, sensory capture (supporting vision and hearing) to brain protection. Diversity of skull function is reflected by its complex and highly variable morphology. Cranial morphology can be quantified using geometric morphometric techniques to offer invaluable insights into evolutionary patterns, ecomorphology, development, taxonomy, and phylogenetics. Therefore, the skull is one of the best suited skeletal elements for developmental and evolutionary analyses. In contrast, less attention is dedicated to the fibrous sutural joints separating the cranial bones. Throughout postnatal craniofacial development, sutures function as sites of bone growth, accommodating expansion of a growing brain. As growth frontiers, cranial sutures are actively responsible for the size and shape of the cranial bones, with overall skull shape being altered by changes to both the level and time period of activity of a given cranial suture. In keeping with this, pathological premature closure of sutures postnatally causes profound misshaping of the skull (craniosynostosis). Beyond this crucial role, sutures also function postnatally to provide locomotive shock absorption, allow joint mobility during feeding, and, in later postnatal stages, suture fusion acts to protect the developed brain. All these sutural functions have a clear impact on overall cranial function, development and morphology, and highlight the importance that patterns of suture development have in shaping the diversity of cranial morphology across taxa. Here we focus on the mammalian cranial system and review the intrinsic relationship between suture development and morphology and cranial shape from an evolutionary developmental biology perspective, with a view to understanding the influence of sutures on evolutionary diversity. Future work integrating suture development into a comparative evolutionary framework will be instrumental to understanding how developmental mechanisms shaping sutures ultimately influence evolutionary diversity.

## An Introduction to Sutures

Cranial diversity is shaped by the unique development and functional complexity of the skull. This diversity reflects vast ecological diversification present across vertebrates (McCurry et al., 2015; Arbour et al., 2019; Felice et al., 2019; Watanabe et al., 2019). The skull performs many functions enabling each species to thrive within their unique ecological niche, by supporting prey acquisition, feeding, and breathing, while protecting the brain and sensory organs to support sensory capture (i.e., vision and hearing). Across vertebrates, the adjacent cranial and facial bones are connected by fibrous joints, known as cranial sutures (Opperman, 2000). As joints, the term “suture” therefore encompasses both the fibrous connective tissue and the osteogenic expanding bone fronts connected by such fibres (Lenton et al., 2005).

Recent instrumental advances in geometric morphometric techniques have supported an extensive body of research considering the comparative morphology, functioning, and development of the skull (Stayton, 2005; Pierce et al., 2009; Brusatte et al., 2011; Foth et al., 2012; Esteve-Altava et al., 2013; Fabre et al., 2014; Lu et al., 2014; Parr et al., 2016; McCurry et al., 2017; Felice and Goswami, 2018; Heck et al., 2018; Bardua et al., 2019; Evans et al., 2019). In contrast, relatively little work focusses on the comparative morphology of cranial sutures (Kathe, 1999; Monteiro and Lessa, 2000; Byron, 2009; Buezas et al., 2017). Even less has work directly addressed the interrelationship between the skull and sutures (Richtsmeier et al., 2006; Heuzé et al., 2010; Esteve-Altava and Rasskin-Gutman, 2015), in particular in a comparative taxonomic framework (Richtsmeier et al., 2006; Heuzé et al., 2010; Esteve-Altava and Rasskin-Gutman, 2015). To highlight this difference, a Google Scholar search returned a 20-fold difference in the number of papers containing the keyword skull compared to those containing the keywords skull and sutures. The limited work addressing comparative suture morphology compared to the comprehensive analysis of cranial morphology, stems, in part, from the inherent natural complexity of sutures. For example, the open outlines of sutures (i.e., sutures do not exhibit a closed shape) can complicate comparative analysis of their morphology (Canfield and Anstey, 1981; Allen, 2006). Moreover, there is a distinct lack of well-defined homologous anatomical landmarks that can implemented for these non-osseous structures (Toussaint et al., 2021).

From an evolutionary perspective, sutures can broadly refer to any connections between hard tissue structures, including those found across both vertebrate and invertebrate clades. The earliest examples of such structures can be found in trilobites from the Cambrian (541–485 Ma) (Paterson and Edgecombe, 2006). Cranial examples, which are not homologous to the earlier examples found in trilobites and other extinct groups such as ammonoids are first observed in fossil fishes from the Ordovician (485–444 Ma) (Bryant, 1932). Such cranial sutures are common to all clades of vertebrates (Kathe, 1999; Clack, 2002; Rayfield, 2005; Markey et al., 2006; Curtis et al., 2013; Rager et al., 2014; Kammerer et al., 2015; Bailleul et al., 2016; Bailleul and Horner, 2016), including both extinct and extant mammals (Goswami et al., 2013; Rager et al., 2014). Whilst the sutures of trilobite exoskeletons and ammonoid shells are clearly not homologous to the cranial sutures of modern vertebrates, some interesting similarities have been proposed. For example, both types of sutures may have been shaped in response to external pressures. It is well known that vertebrate suture morphology responds to stresses imposed by behaviour and ecology (Jaslow and Biewener, 1995; Clack, 2002; Goswami et al., 2013; Buezas et al., 2017). It has similarly long been postulated that ammonoid shell suture morphology may also respond to ecological stresses such as hydrostatic pressure (Hewitt and Westermann, 2007), although there is controversy within the literature and this idea is still debated (Lemanis, 2020). Remarkably, these non-homologous sutures may have convergently evolved to respond to similar external pressures.

**FIGURE 1:**
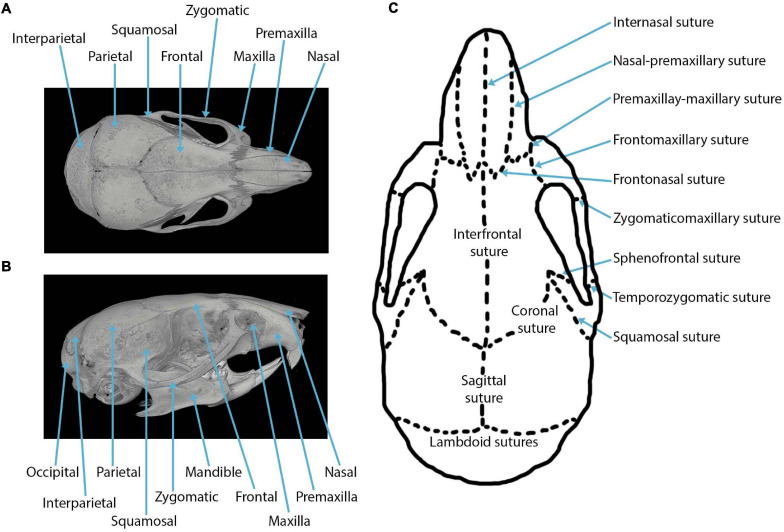
Craniofacial anatomy: **(A)** dorsal view of a mouse skull depicting the cranial bones in a microCT reconstruction; **(B)** lateral view of a mouse skull depicting the cranial bones in a microCT reconstruction; **(C)** cranial sutures in the dorsal view.

The major sutures of the mammalian cranial vault (Figure 1) form between: the paired frontal bones to create the interfrontal (metopic) suture; between the paired parietal bones to create the sagittal suture; between both frontal and parietal bones to form the paired coronal sutures; between the interparietal and two parietal bones to form the paired lambdoid sutures; and between both squamosal and parietal bones to form two squamosal sutures (shown in the mouse in Figure 1). Three sutures separate the neurocranium from the viscerocranium (Figure 1): the frontonasal suture forms between the nasal bone of the viscerocranium and the frontal bone of the neurocranium; the frontozygomatic suture forms between the zygomatic bone of the viscerocranium and the frontal bone of the neurocranium; the temporozygomatic suture forms between the zygomatic bone of the viscerocranium and the squamosal bone of the neurocranium (Hanken and Hall, 1993). The presence of these cranial sutures supports skull function.

It is worth noting here that not all joints of the skull are sutures. Sutures, as mentioned above, are fibrous joints forming between the membranous bones of the skull, whereas synchondroses form cartilaginous joints between the endochondral bones of the skull (Opperman et al., 2005). Whilst synchondroses differ developmentally from sutures, they function in a similar manner to facilitate the growth of cranial bones and thus have crucial roles during postnatal craniofacial development (Cendekiawan et al., 2010). In humans, synchondroses are recognised to be functionally important for enabling cranial base flexion to accommodate the increased brain volume (encephalization), whilst cranial base flexion in turn influences facial projection (Lesciotto and Richtsmeier, 2019). It is important to highlight here that many evolutionary studies commonly refer to synchondroses as sutures and thus use the term suture to broadly include both fibrous and cartilaginous joints.

Whilst sutures are present in the skulls of all vertebrates, suture development, morphology, complexity, and fusion patterns differ both within and across species. The phylogenetic distance between clades (e.g., birds and mammals) has resulted in alternative mechanisms of suture growth and fusion. For example, archosaurs have a greater diversity in the number of sutures present than mammals, in addition to using an entirely different mineralised tissue at the sutures themselves (Bailleul and Horner, 2016). Therefore, focussing on one vertebrate clade minimises comparison challenges, such as differences in suture number and structure, allowing direct analysis of how suture morphology influences cranial morphology. Mammals are an optimal clade to study in order to understand the drivers of morphological variation and diversification due to their broad ecological diversification into a range of habitats on every continent and in every ocean, coupled with their variation in both cranial and suture morphology. Moreover, the mouse model is one of the most commonly used model systems within developmental biology research, meaning that applicable developmental data is readily available. Despite the variation in suture shape, the composition of the mature suture in mammals appears to be largely comparable across various mammalian species (Pritchard et al., 1956; Persson et al., 1978; Bailleul and Horner, 2016). Therefore, suggesting that the process and general principles of postnatal suture development are similar across mammalian taxa and that lessons from one species could be applied to others. This conserved structure could dictate the similar functioning of sutures across mammals, such as providing shock absorption (Moazen et al., 2009) and supporting mastication (Goswami et al., 2013). Collectively, mammals present an ideal system for studying the frontier between evolutionary and developmental biology.

Here, we review current knowledge on the interaction between suture development and morphology and cranial shape in mammals from an evolutionary developmental biology perspective, with a view to understanding suture development and its influence on evolutionary diversity. From several perspectives, it is clear that suture closure plays a role in shaping human cranial morphology. Pathology research suggests that the order of cranial suture fusion is instrumental in producing various craniofacial morphologies (Heuzé et al., 2010). Anthropological researchers have proposed the hypothesis of functional craniology (Moss and Young, 1960; Bruner, 2007) which has led to the notion that sutures are themselves crucial elements of the cranial functional network (Di leva et al., 2013). Theoretical morphological studies, implementing network models of the skull, have also highlighted the potential importance of suture closure timing in shaping the human skull form (Esteve-Altava and Rasskin-Gutman, 2015). Combined, these studies and others suggest a relationship between cranial form and suture morphology, fusion, and functioning. We have, therefore, compiled evidence from human pathology, mouse mutants, comparative anatomy, and evolutionary trends, to further develop our understanding of this interaction between suture development and morphology and mammalian cranial shape.

## Suture Morphology

Suture morphology has been studied on a number of levels, covering the microscopic morphological organisation of the fibres, the cross-sectional joint morphology, and the gross morphology spanning the entire sutural length. It is well known that suture morphology and fusion adapt over time by responding to a number of pressures (Wilkie and Morriss-Kay, 2001; Clack, 2002; Byron, 2009; Buezas et al., 2017). Here, we refer to “suture fusion” as the closure of the suture, which is accompanied by the process of suture obliteration, whereas the term “patent suture” describes the sutures remaining open and unfused.

At the microscopic level, a dense network of Sharpey’s fibres creating a matrix of connective tissue, consisting mainly of type 1 collagen, forms the fibrous sutural joint connection between two approaching bone fronts (Pritchard et al., 1956; Khonsari et al., 2013). The microscopic organisation of these fibres has been studied using various methods including confocal laser scanning, histology, and synchrotron X-ray microtomography, all of which identify a unique fibre orientation pattern for each suture locale. Sutures undergoing fusion, such as the interfrontal suture in the mouse, have been identified to have a highly organised lattice of Sharpey’s fibres with new bone matrix deposition (Koskinen et al., 1976; Warren et al., 2008; Khonsari et al., 2013). In contrast, sutures that remain patent, such as the sagittal suture, have a random arrangement of fibres (Warren et al., 2008). In scenarios of a convex approaching bone front, fibres have been observed to present in a fan pattern (Khonsari et al., 2013). This variable fibre orientation across the sutures appears to be associated with minute growth, specific to the suture locale, thus producing a unique microscopic morphology at each suture (Koskinen et al., 1976). Moreover, the orientation of these Sharpey’s fibres adapts throughout ontogeny creating a pattern that is specific to the growth at the suture locale (Koskinen et al., 1976). Over time, the fibrous connective tissue becomes increasingly organised, forming straighter collagen fibrils connecting the approaching bones (Zimmermann et al., 1998). At this microscopic level, a unique morphology can evidently be observed at each suture location.

**FIGURE 2:**
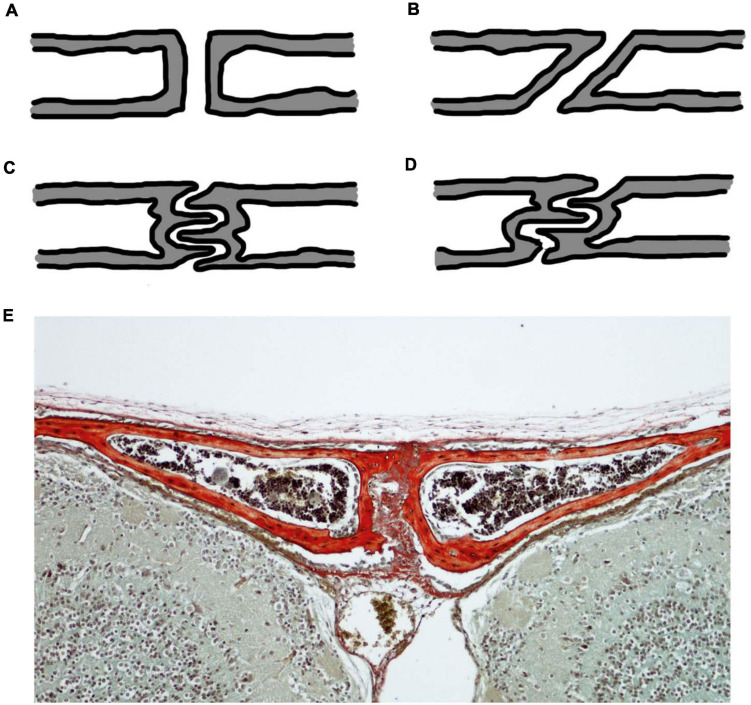
Cross-sectional suture joint morphology: **(A)** butt joint; **(B)** bevelled joint; **(C)** finger joint at a butt end-to-end connection; **(D)** finger joint at an overlapping bevelled connection; **(E)** CD1 mouse trichrome stain at P20 showing the cross-section through the interfrontal suture with bone indicated in red reflecting the butt joint in **(A)**. Schematics **(A–D)** based on (Moss, 1957; Koskinen et al., 1976).

Akin to any other mechanical joint, sutures present with different joint types (Figure 2). For sutures, the joint type is identified in the cross-section through the suture. In this cross-section, butt joints form end-to-end connections (Figures 2A,E), bevelled joints present with overlapping bone fronts (Figure 2B), and finger joints have interlocking and interdigitations (Figures 2C,D; Moss, 1957; Koskinen et al., 1976). Consequently, interdigitations are not a unique feature of gross suture morphology, but also exist across internal suture cross-sections, suggesting that complexity might be hidden from a superficial gross morphological view. The type of sutural joint is not thought to be predetermined, but instead thought to occur as a consequence of mechanical pressure from functional demands (Moss, 1957; Herring, 1972; Koskinen et al., 1976). Therefore, it is unsurprising that sutures start out as the simplest joint type, end-to-end joints, and throughout ontogeny develop to include other joint types which are modifications of this simple butt joint morphology (Moss, 1957).

**FIGURE 3:**
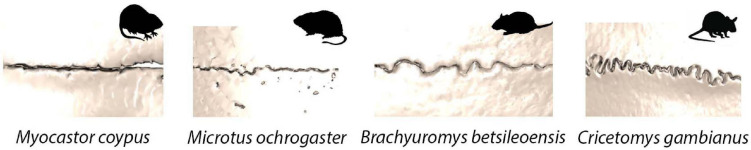
Sagittal suture morphological variation in four species of rodent: *Myocastor coypus* (coypu); *Mircotus ochrogaster* (prairie vole); *Brachyuromys betsileoensis* (Betsileo short-tailed rat); *Cricetomys gambianus* (Gambian pouched rat).

The gross morphological scale captures the entire suture length and is outlined by the approaching bone fronts. At this gross scale, suture morphology is once again highly variable, with the same suture having a very different morphology even in relatively closely related species of mammal (Figure 3). Patterns span a range of morphologies, including straight, highly curved, looping, and interdigitated outlines (White et al., 2020). Interestingly, there is a strong association between the microscopic fibre organisation and the gross suture morphology (Khonsari et al., 2013). Mechanical constraints and pressures generated during sutural growth are thought to influence the fibre orientation which in turn shapes and modifies the developed gross suture morphology (Khonsari et al., 2013). Throughout ontogeny, gross suture morphology undergoes large morphological transformations, starting out as straight morphologies and developing to become highly interdigitated with complex patterns (Nicolay and Vaders, 2006; Curtis et al., 2014). In the mouse, such interdigitations are established at around 7 weeks postnatal, following sexual maturity and progress as growth continues to adulthood (3 months) (Miura et al., 2009).

In the developed suture, gross morphology varies between the sexes, from suture to suture, and across species. Sexual dimorphism of cranial sutures has been identified for certain species. Specifically, male wild sheep (*Ovis orientalis*) exhibit a greater degree of complexity in the facial sutures than females, which is thought to be a result of head-to-head fighting (Jaslow, 1989). Additionally, sutures in different cranial regions have been reported to have differing morphologies, with straight sutures identified in the facial region and interdigitated sutures within the braincase (Monteiro and Lessa, 2000). Finally, the level of sutural interdigitation, ranging from low to high, reflects interspecific variation at different taxonomic levels, within both genus (*Cebus*) and infraorder (Caviomorpha) (Byron, 2009; Buezas et al., 2017). Interspecific suture morphological variation likely implies the presence of heightened developmental variation. Several metrics have previously been proposed to quantify this gross morphological complexity of sutures, which have recently been compared on mammal sutures (White et al., 2020). Quantification of gross suture morphological complexity will enable the untangling of mechanisms driving the alterations in complexity. Nevertheless, this is complicated by the number of external factors involved in shaping suture morphology. Factors driving this gross suture variation have been attributed to a number of biological pressures, such as diet, behaviour, and ecology (Jaslow and Biewener, 1995; Herring and Teng, 2000; Monteiro and Lessa, 2000; Byron et al., 2004; Byron, 2009; Buezas et al., 2017), in addition to patterns of growth (Henderson et al., 2005) and trends toward increasing complexity over geologic time scales (Allen, 2006). Irrespective of the scale at which suture morphology has been studied (micro, cross-sectional, gross), a unique morphology specific to the suture locale can be observed. Whilst it is possible that suture phenotypic variation could be used to interpret information regarding the external stresses, deriving the specific pressure responsible for a suture phenotype, however, is difficult given the complexity of factors and stresses (development, sex, diet, ecology, and behaviour) proposed to be involved. As studies have only been conducted on a limited range of mammalian taxa to date, as discussed above, the need for comparative studies becomes ever more pertinent to help reveal evolutionary differences and stresses involved in shaping the suture at the micro, cross-sectional and gross morphological scales.

## The Importance of Sutures

As the major joints of the skull, sutures have a number of crucial functional roles. Each role supports the overall functioning of the cranium, highlighting a functional link between sutures and the skull.

Open sutures act as signalling centres to regulate the balance between proliferation of osteoblast precursors and osteogenic differentiation (Iseki et al., 1997; Zhou et al., 2000). Consequently, sutures are able to carry out one of their key functional roles, by acting as the major site of interstitial bone growth for the cranial bones of the skull (Baer, 1954; Opperman, 2000; Lana-Elola et al., 2007; Jin et al., 2016). During postnatal development, the suture mesenchyme provides a unique niche operating as a reservoir for mesenchymal stem cells (MSC), which are key to supporting cranial bone growth. Within the suture mesenchyme, lineage tracing experiments have identified Gli1+ cells to be the main MSC population (Zhao et al., 2015). These Gli1+ cells function to support the growth of all craniofacial bones (Zhao et al., 2015). Moreover, an Axin2 expressing cell population, identified more specifically within the midline suture mesenchyme, has also been associated with calvarial development (Maruyama et al., 2016). A third population of similarly located Prx1 positive cells has also been recently identified (Wilk et al., 2017). As such, MSC populations within the suture mesenchyme enable the cranial bones to expand in a coordinated manner around the growing brain and can act as a reservoir of cells during homeostasis. Evidence for this interstitial bone growth role across evolutionary taxa, has been identified outside of mammals. In the zebrafish, studies suggest that patency is also necessary for maintaining a stem cell population at the suture locale required for cranial bone osteogenesis (Teng et al., 2018). Beyond bone growth, sutures also support cranial bone repair by providing a major source of stem cells (Doro et al., 2017). While the Gli1 population has been shown to have a role in homeostasis and repair (Zhao et al., 2015), the Axin2 population appears to represent a reserve population specifically activated during injury (Maruyama et al., 2016). Therefore, craniofacial bone homeostasis seems to be highly dependent upon this unique niche of sutural mesenchymal stem cells. It is possible, with much future work, that these stem cell populations could be harnessed to support craniofacial repair in patients, through the design of new therapies for future clinical implementations (Doro et al., 2017). Once closed, sutures appear to lose their MSC population. The Gli1+ population is lost at the site of the fused interfrontal suture in mice (Zhao et al., 2015), whilst there is a reduction in the Axin2 population at prematurely fused sutures in craniosynostosis patients (Di Pietro et al., 2020). Thus, suggesting that an open suture status is necessary for the suture to contain a reservoir of MSCs enabling it to function as a site of interstitial bone growth and support normal craniofacial development.

Sutures additionally function to allow skull movement. During birth, in humans, the sutures enable movement of the cranial bones, creating overlapping to ease the passage through the birth canal (Jin et al., 2016). This functional role of the sutures is likely to have greater significance in placental mammals than in monotremes or marsupials, since the short gestation period results in highly altricial young (Lillegraven et al., 1987; Nunn and Smith, 1998). Marsupials exhibit a lower level of closure than placentals, and sutures are reported to remain open throughout life (Rager et al., 2014). It is thus possible that variation in suture closure between placentals and marsupials may be indicative of differing postnatal functional roles, especially given the significance of suture fusion status in supporting postcranial bone growth (Zhao et al., 2015; Di Pietro et al., 2020).

Fused sutures, as well as open sutures, are both functionally important. Across placental mammals, sutures of the cranial vault and synchondroses of the cranial base generally close postnatally (Rager et al., 2014). Suture fusion provides protection to the developed brain. Interestingly, the sutures of the facial region of most placentals never fuse (Rager et al., 2014) and suggest additional functional roles for open sutures, such as an involvement in the masticatory process (Herring, 1972; Flores et al., 2006; Goswami et al., 2013). A modelling approach utilising finite element analysis (FEA) has indicated that stresses are mitigated in skulls with a higher number of open sutures, suggesting that sutures are essential for shock absorption and the redistribution of strain across the skull (Moazen et al., 2009). Therefore, strains that are produced during biting can be modified across the skull as a result of suture presence (Moazen et al., 2009). Strain at the suture is both greater than within the adjacent cranial bones and is dependent on the muscle activity during mastication (Herring and Teng, 2000). Moreover, sutures facilitate the process of feeding by providing compliant and elastic joint mobility within the skull (Herrel et al., 2000; Herring and Teng, 2000). In geckos, cranial kinesis, which is supported by the cranial sutures, offers the capability for a larger bite force (Herrel et al., 2000). It is possible this functional role of sutures observed in cranial kinesis of geckos is mimicked in mammals which also exhibit cranial kinesis, such as rabbits (Kraatz and Sherratt, 2016). Not only are sutures functionally important during feeding, but they also act as shock absorbers to absorb strains from other external inputs, such as fighting behaviours and locomotion (Curtis et al., 2013). Evidently, sutures have hugely important functional roles both across postnatal development and throughout an organism’s life, all these roles support and contribute to the normal functioning of the skull.

The importance of proper suture functioning for maintaining normal craniofacial development across postnatal development is evidenced in examples of abnormal suture development and when sutural defects occur. Craniosynostosis describes a premature pathologic fusion of one or more sutures and was first coined by Otto (1830). The prevalence of craniosynostosis is estimated to be between 1 in 2,000 and 1 in 2,500 live births (Johnson and Wilkie, 2011), with an increasing rate of occurrence reported (Cornelissen et al., 2016). Molecular and developmental studies have highlighted the complex pathogenesis that underlines craniosynostosis and how this contributes to the multitude of craniofacial dysmorphologies arising from premature suture fusion (Flaherty et al., 2016).

Craniosynostosis can either present as syndromic or non-syndromic. It can also be classified as either primary or secondary (developmental disorder directly or indirectly targeting the suture), and simple or compound (one or multiple sutures involved) (Flaherty et al., 2016). Syndromic craniosynostosis is associated with a genetic abnormality which in turn interrupts various signalling pathways to produce a number of dysmorphologies. Of these dysmorphologies one of the defects is craniosynostosis (suture fusion), resulting in craniofacial abnormalities (Senarath-Yapa et al., 2012). Numerous mutations, disrupting a multitude of signalling pathways, have been associated with craniosynostosis (Flaherty et al., 2016). In contrast, non-syndromic craniosynostosis is the fusion of a cranial suture in the absence of trunk, limb or other dysmorphologies; it is the suture fusion that is the most pronounced phenotypic abnormality (Flaherty et al., 2016). Examples of syndromic craniosynostosis include: Antley-Bixler syndrome, Apert syndrome, Carpenter syndrome, Crouzon syndrome, Muenke syndrome, Pfeiffer syndrome, and Saethre-Chotzen syndrome (Wilkie and Morriss-Kay, 2001; Lenton et al., 2005). Of these syndromes, Muenke syndrome is the most common and presents with coronal suture synostosis (O’Hara et al., 2019). However, in non-syndromic craniosynostosis the sagittal suture as the most frequently affected (Kimonis et al., 2007; Flaherty et al., 2016). Aside from the coronal and sagittal sutures, the interfrontal and lambdoid sutures are also commonly affected (Kimonis et al., 2007). In each of these examples, craniosynostosis of the suture has a resultant effect on the cranial morphology (Flaherty et al., 2016).

Morphological changes in the skull can also impact on the brain. In severe cases, brain expansion becomes limited or distorted from the alterations in cranial morphology leading to cognitive deficits (Shim et al., 2016). The reduction in cranial size induces a physiological increase in intracranial pressure, which creates a number of functional impairments, manifesting as visual impairments, deafness, and further cognitive deficits (Gault et al., 1992; Panchal and Uttchin, 2003), requiring surgical correction (Lenton et al., 2005; Slater et al., 2008). Craniosynostosis reveals a persistent co-adjustment between the brain and skull, which, with advancing research will better inform surgical interventions and more accurately predict outcomes (Lesciotto and Richtsmeier, 2019). More detail on premature suture fusion, the associated signalling pathways, and the resultant craniofacial dysmorphologies (craniosynostosis) are discussed in greater detail later in this review in order to consider whether sutures may act as targets for evolutionary change in cranial morphology. Nevertheless, it is evident that correct suture function is critical for normal craniofacial development and functioning.

## Suture Development

Understanding normal suture development is pertinent to our understanding of the significance of abnormal suture development from a pathological perspective. Moreover, an understanding of suture development allows for an appreciation of how developing sutures can act as targets for evolutionary change. Suture development has been studied using a wide array of animal models, including the mouse, rat, rabbit, sheep, frog, and zebrafish (Grova et al., 2012). Comparisons across these distantly related vertebrate clades can be complicated by variation in developmental timings, origins, and processes (Bailleul and Horner, 2016). Here, we focus on mouse sutural development, as murine sutures have been shown to share major similarities with other mammalian taxa, including humans (Grova et al., 2012; Rager et al., 2014), thus a considerable amount of work is available documenting mouse craniofacial development making it an ideal model system for studying suture development from a pathological perspective (Wilkie and Morriss-Kay, 2001). Moreover, given the similarities in mature suture composition across mammals, information pertaining to mouse suture development and function will also be useful for understanding the pattern of suture formation, fusion, and thus functioning in mammals (Pritchard et al., 1956; Bailleul and Horner, 2016). Where other animal models are integrated within the discussions below, this will be specified. For the purpose of this review, focus will be given to the development of the sutures within the cranial vault, due to the importance of cranial vault sutures in facilitating brain growth. More detailed discussions of suture development can be referred to in the literature (Opperman, 2000; Lenton et al., 2005; Flaherty et al., 2016).

**FIGURE 4:**
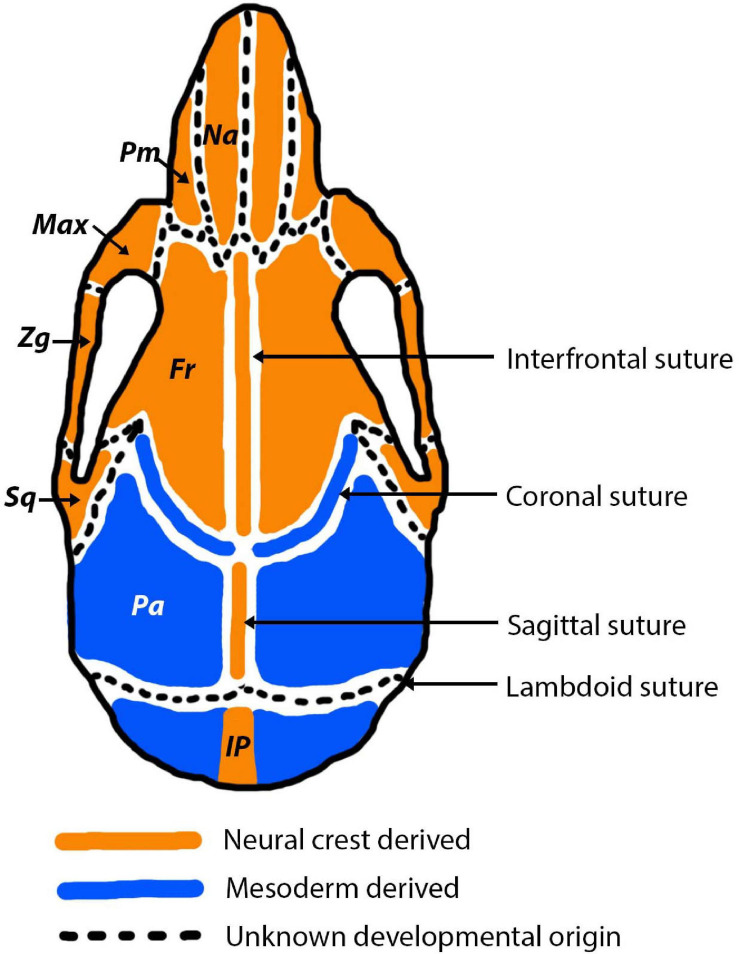
Cranial bone and cranial suture developmental origins pictured in the adult mouse. Neural crest derived bones and sutures are depicted in orange and mesodermally derived bones and sutures are depicted in blue. In the early stages of development (E15.5-P0) the origins of the sutures are more nuanced, for example the sagittal and coronal sutures express a combination of mesoderm and neural crest derived cells (Doro et al., 2019). Abbreviations are as follows: Na, nasal; Pm, premaxilla; Max, maxilla; Zg, Zygomatic; Sq, squamosal; Fr, frontal; Pa, parietal; IP, interparietal.

During early embryogenesis, the mesenchyme that forms the basis for the vertebrate fetal head is derived from two different developmental origins, mesoderm and neural crest (Couly et al., 1993; Jiang et al., 2002; Le Douarin, 2012). The neural crest originates from the ectoderm and undergoes an epithelial-mesenchymal transition to form a migratory population of mesenchymal cells (Hay, 2005). The developed skull can be separated into the viscerocranium and the neurocranium. Aspects of each of these are derived from different developmental origins (Figure 4). The viscerocranium, which makes up the bones of the facial region, is produced exclusively by neural crest derived cells (Le Douarin, 1982; Jiang et al., 2002). In mammalian development, the cranial neural crest cells are localised in the first and second pharyngeal arches, which contribute to the formation of the viscerocranium (Chai et al., 2000). In contrast, the neurocranium, which includes the cranial regions protecting the brain (cranial vault and base), has been strongly debated in terms of its embryological origins. Avian developmental studies have opposingly identified that the neurocranium is derived either solely from neural crest (Couly et al., 1993), or from a combined neural crest and mesoderm origin (Le Lièvre, 1978; Noden, 1988). More recently, increasingly precise labelling techniques agree that the neurocranium is formed from both neural crest and mesoderm, with the frontal bone being of dual origin and the remainder of the neurocranium mesodermally derived (Evans and Noden, 2006). Mammalian developmental studies agree with the vast majority of avian research, which suggests that the neurocranium is comprised of bones derived from both neural crest and mesoderm origins (Jiang et al., 2002; McBratney-Owen et al., 2008; Le Douarin, 2012). The neurocranium can be subdivided into the cartilaginous aspect (chondrocranium) and the membranous aspect (dermatocranium), both of which consist of bones from both cellular origins. Lineage tracing experiments in the mouse using a Wnt1-Cre allele and Mesp1-Cre allele, to mark neural crest and mesoderm origins, respectively, have shown the that the neural crest-mesoderm interface lies within the neurocranium (Jiang et al., 2002; Yoshida et al., 2008; Maddin et al., 2016; Teng et al., 2019). This boundary falls between the neural crest derived frontal bones and the mesoderm derived parietal bones (Figure 4).

Sutures of the murine cranial vault (interfrontal, sagittal, coronal, lambdoid) often form directly at the interface between mesoderm and neural crest derived tissue (Jiang et al., 2002; Lenton et al., 2005). Not only do the sutures separate bones of differing embryological origin, but they are themselves derived from different origins (Figure 4). The neural crest-mesoderm boundary separating the neural crest derived frontal bones and mesodermal derived parietal bones in the mammalian skull has been pinpointed to the coronal suture (Maddin et al., 2016; Teng et al., 2019). Moreover, the mesenchyme of the coronal suture itself is of mesoderm origin, which means it forms a direct neural crest-mesodermal interface (Jiang et al., 2002). This interface is established in early stages of development, at E9.5, and later leads to the formation of the coronal suture (Jiang et al., 2002). In contrast, the sagittal suture separates the two mesodermal parietal bones, but creates a neural crest-mesoderm boundary as the suture mesenchyme is neural crest (Jiang et al., 2002; Lenton et al., 2005). As a result of the neural crest-mesoderm boundaries, it has been speculated that the sagittal and coronal sutures have the greatest contributions to cranial growth, due to being present at the interface between the two tissue origins (neural crest and mesoderm) (Jiang et al., 2002). In contrast, the interfrontal and lambdoid sutures are not thought sit at an interface, but instead separate the neural crest derived frontal bones and the mesoderm derived parietal and interparietal bones, respectively. However, the origin of the lambdoid suture, like with many others, is unknown and therefore it is unclear if the lambdoid suture does sit at a neural crest-mesoderm interface (Lenton et al., 2005).

Following the appearance of a neural crest-mesoderm interface evident at E9.5 (Jiang et al., 2002), a cranial bone matrix is established at around E15 (Alberius and Friede, 1992). The cranial bones begin to grow and expand toward each other via both intramembranous and endochondral ossification, dependent on the bone. This process has been reported after 2 days of culture of E15.5 parietal bones and at E19 in the rat (Kim et al., 1998; Opperman, 2000). At this stage, each bone is widely separated by mesenchyme also referred to as the presumptive sutural matrix (Opperman et al., 1993; Opperman, 2000). Presumptive sutural matrix is formed at specific locales across the developing skull. For the coronal suture, as mentioned previously, this develops directly at the interface between neural crest and mesoderm (Jiang et al., 2002), whereas the other sutures of the cranial vault, form at anatomical landmarks of the underlying brain tissue. For example, the sagittal and interfrontal sutures develop at the midline between the cerebral hemispheres and olfactory lobes and the lambdoid suture develops between the cerebral hemispheres and the cerebellum (Morriss-Kay and Wilkie, 2005).

A significant part of suture development in the mouse occurs postnatally. After birth, the presumptive sutural matrix creating the gap between the approaching parietal bone fronts, at the location of the sagittal suture, distinctly reduces (P1) (Zimmermann et al., 1998). Within the mesenchyme of this presumptive sutural matrix the presence of blood vessels is evident. At this stage, a fibrous structure forms at the prospective mineralisation sites for the parietal bones. During the early stages of postnatal development of the suture, there is a clear a shift with the underlying mesenchyme forming fibrous connective tissue separating the bone margins (Markens, 1975; Zimmermann et al., 1998; Lenton et al., 2005). At P9 a homologous collagen matrix replaces the presumptive sutural matrix and forms a straight fibrous connective tissue by P14 (Zimmermann et al., 1998). The parietal bone plates surrounding the sagittal suture undergo a large thickening at the later stages of development (P14–20). As cranial expansion slows, at around P20 (P21 in the rat), following a reduction in the number of cells lining the bone fronts, the sutures become increasingly narrow to create mature sutures. At the mature suture, the previously approaching bones are connected by collagen fibrils of the fibrous connective tissue (Zimmermann et al., 1998; Opperman, 2000). The various sutures reach a matured state at different postnatal ages, variability is also observed across species. In the rat, as cranial expansion slows by P21, the coronal suture narrows to reach its fully formed developed state (Opperman et al., 1993). In the mouse, the sagittal suture forms by P20 and reaches maturity by P26–28 (Zimmermann et al., 1998). At these final stages of postnatal development, the collagen fibrils run continuously between the fibrous connective tissue of the suture into the bone to create a highly organised cross-striated bone structure (Zimmermann et al., 1998). As cranial expansion slows (P20–28) once the fibrous connective tissue of the sutures has formed, sutures become the primary site of craniofacial osteogenesis (Opperman, 2000; Luo et al., 2019). As the mature suture composition is similar across mammals (Pritchard et al., 1956; Persson et al., 1978; Bailleul and Horner, 2016), developmental information from the mouse is likely to be applicable across mammalian species and could result in similar suture functioning (Herrel et al., 2000; Moazen et al., 2009; Curtis et al., 2013; Goswami et al., 2013).

Once formed, the decision to keep a suture open (patent) or closed is essential for the coordinated growth of the cranial bones and brain. In the mouse, patency is seen throughout life for many sutures, including the sagittal, coronal and lambdoid sutures (Lenton et al., 2005). However, not all sutures remain patent but instead undergo fusion in order to protect the developed brain. The only suture to undergo such fusion in the mouse is the interfrontal suture which fuses at around P7–12 (Bradley et al., 1996), occurring in the early stages of postnatal development prior to the point of sexual maturity. Across different species there appears to be a large degree of variation as to which sutures fuse and which remain patent (Rager et al., 2014). Interestingly, whilst the fusion of the interfrontal suture in mice is akin to that of humans, all other sutures in the human skull fuse in adulthood unlike the mouse (Weinzweig et al., 2003). Nevertheless, throughout the process of postnatal development sutures narrow, progressing from an open to near fused or fused status.

The commonality of suture fusion occurring in the late stages of postnatal skull development unites mammals and thus the mouse model in terms of their postnatal suture development (Rager et al., 2014; Esteve-Altava et al., 2020), and contrasts with some species of reptile where the sutures are thought to become increasingly patent across ontogeny (Bailleul et al., 2016). As suture patency is key to suture function, extending the life of an open suture would maintain the reservoir of mesenchymal stem cells at the suture enabling it to act as a site of bone growth, homeostasis, and repair for longer (Zhao et al., 2015), as well as providing shock absorption for longer across life (Moazen et al., 2009). Such functional roles are likely applicable across mammals, given that sutural stem cell populations necessary for interstitial cranial bone growth have been identified beyond mammalian species (Teng et al., 2018). Therefore, the transition of a suture from an open to a fused status, or vice versa, has the potential to impact the shape, size, and function of the associated cranial bones, which as a consequence could be a driver for evolutionary skull morphological change and variation across mammals.

## What Can We Learn From a Suture?

**FIGURE 5:**
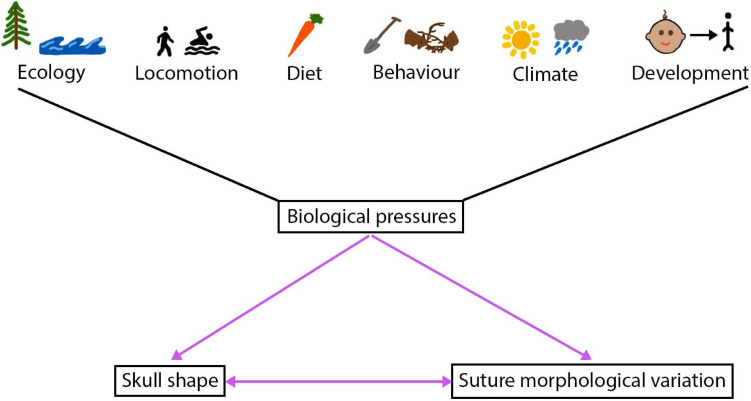
The intertwined relationship between the cranial sutures and skull, a number of biological pressures shape this relationship.

Beyond their crucial roles during development and their functional importance, sutures are becoming increasingly powerful for understanding species ecology and life history across a number of vertebrates (Figure 5). As noted above, suture fusion and morphology are thought to respond to biomechanical stresses from the external environment, suggesting that sutural features and morphology may aid in the interpretation of functional pressures driving cranial form. The influence mechanical pressures have on shaping sutures, where external forces alter sutures from a butt joint morphology to a bevelled or finger joint or create a gross interdigitated morphology, means it may be possible, in the future, to interpret the diet, behaviour, ecology and mechanical stresses of an individual from the suture morphology (Moss, 1957; Oudhof, 1982; Kathe, 1999; Monteiro and Lessa, 2000; Clack, 2002; Nicolay and Vaders, 2006). As the complexity of mammal suture morphology is also thought to respond to biological pressures (Herring, 1972; Nicolay and Vaders, 2006), advances in morphometric techniques make it is possible to quantify and compare suture morphological complexity across a range of mammalian species (White et al., 2020). As such, we may be able to use suture morphology to better infer organismal biology and morphology alone. Current work has identified that harder diets and chisel-tooth digging of caviomorph rodents place greater demands on the sutures, thus increasing suture complexity (Buezas et al., 2017). In the rodents shown in Figure 3, the most complex suture is observed in *Cricetomys gambianus* (Gambian pouched rat). The differences seen here in suture complexity are likely to be due, in part, to the differences in muscle mass at the jaw joint in the different species, with larger muscles producing a higher bite force and in turn generating an increasingly complex suture morphology (Byron et al., 2004). Interestingly, the connective tissue within the mammalian sutural joint itself is also known to respond to biomechanical pressures, suggesting that the micro-scale morphology could similarly be used to shed light on localised functional pressures (Byron et al., 2004).

Suture closure patterns in addition to suture morphology, at all scales (macro/meso/micro), could similarly be used to understand external stresses acting on a species. Suture closure patterns have been reported to indicate locomotory strategies in several species of Hystricognathi rodents (Wilson and Sánchez-Villagra, 2009). Species of Hystricognathi rodents that presented with the highest level of overall suture closure did not follow the pattern of closure proposed by Krogman (1930) which is considered to be generally applicable to mammals (Chopra, 1957). The Krogman pattern of suture closure is as follows: vault, base, circum-meatal, palatal, facial, and cranio-facial. It is these species of Hystricognathi, deviating from the Krogman pattern of suture closure and exhibiting a greater level of suture closure, that displayed different locomotory patterns: sub-terranean (*Bathyergus suillus* and *Nannospalax ehrenbergi*) and arboreal (*Coendou spinosus*, *Coendou insidiosus*, and *Coendou prehensilis*). Suture fusion and morphological complexity appear to provide useful tools for interpreting external pressures, as a result of distortion occurring at the suture to such stresses.

Aside from the ability to shed light on external stresses, sutures can prove useful in understanding developmental timings, growth, ontogeny, and even be used to inform forensic and archaeological dating. During human childhood development, suture development and fusion status are used to follow development and growth (Goyal, 2020). Therefore, an in depth understanding of sutures would further assist in the medical ability to monitor normal cranial development. Sutures can also offer insights into growth rates and craniofacial growth patterns. Bone overlapping occurring within the cross-sectional plane of a suture has been reported to be associated with the growth rate of an individual, using the rat as a developmental model here, with overlapping bones reflecting periods of fast growth (Koskinen et al., 1976). Similarly, in Dinosauria, sutures can provide information about ontogeny, whereby fusion status is considered to be a good proxy for age (Sampson et al., 1997; Fujiwara and Takakuwa, 2011; Longrich and Field, 2012). Additionally, within the fields of archaeology and forensics, suture closure timings have been proposed as a viable option for determining age in humans (Key et al, 1994; Vodanović et al, 2011; Wood, 2015; Ubelaker and Khosrowshahi, 2019). Not only do multiple parameters pertaining to sutures have the capability of providing useful information and shedding light on the fields of evolutionary and developmental biology, but the possibilities extend far beyond these disciplines.

## Sutures as Targets for Evolutionary Change in Skull Morphology

An abundance of information can evidently be taken from the morphology, complexity, fusion pattern, and development of sutures, without beginning to consider the impact such suture parameters have on mature cranial morphology. Nevertheless, suture functioning, development and fusion timing have been highlighted as crucial for supporting normal skull functioning, suggesting a key link between sutures and the skull, as discussed previously. This interdependence is further evidenced by alterations to suture fusion, morphology, and development which cause knock-on changes affecting the cranial form, thus pointing toward an integrated evolution of sutures and craniofacial morphology. Such a relationship, between sutures and skull form, can be explored across several scientific fields on multiple levels, from pathologies, to mouse model systems, to natural variation.

Given the importance sutures have in maintaining the normal development of craniofacial form, many questions arise surrounding what impact the timing, activity, and development of sutures has on mature skull morphology. Evidence from pathology is instrumental in untangling this relationship between sutures and skull shape, as abnormal suture development is often coupled with alterations to the craniofacial morphology. Premature suture fusion is known to result in craniofacial developmental defects, such as craniosynostosis; here, the cranial morphology is distorted in response to early suture fusion (Lenton et al., 2005; Flaherty et al., 2016). More specifically, suture fusion characterised by craniosynostosis causes an excessive cranial bone growth in the parallel axis and prevents expansion in the perpendicular axis, referred to as Virchow’s concept (Virchow, 1851; Delashaw et al., 1999). Consequently, premature sagittal suture fusion produces a long and narrow skull morphology (scaphocephaly), whereas premature coronal suture fusion results in a broad and short cranial morphology (brachycephaly).

**FIGURE 6:**
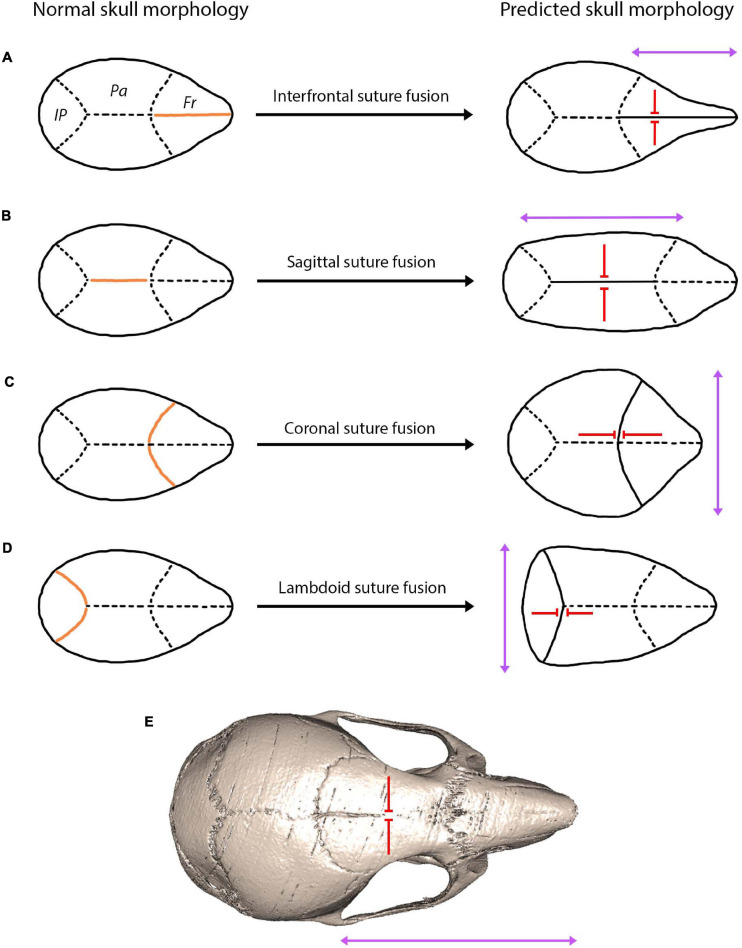
A hypothesis showing the predicted skull morphology following suture fusion, based on the concept of growth occurring in the parallel axis to the fused suture with prevention of growth in the perpendicular axis (Virchow, 1851; Delashaw et al., 1999). Orange indicates the fused suture location, purple arrows indicate the direction of growth, red arrows indicate the direction growth is restricted: **(A)** fusion of the interfrontal suture produces a narrow and elongated skull in the facial region; **(B)** fusion of the sagittal suture produces a narrow and elongated skull in the cranial vault region; **(C)** fusion of the coronal suture produces a shorter and widened cranial vault; **(D)** fusion of the lambdoid suture produces a shorted and widened posterior cranial vault; **(E)** fusion of the interfrontal suture in the mouse using microCT indicates a narrow and elongated facial region similar to the prediction of **(A)**.

Interestingly, finite element analysis has been found to predict skull form following cranial reconstruction of clinically observed craniosynostosis (Malde et al., 2020). This modelling approach has huge implications for understanding craniofacial reconstruction techniques and thus for enhancing clinical remodelling surgery of craniosynostosis patients. Moreover, predictions based on Virchow’s theory on suture fusion patterns and advances in modelling approaches could be used to understand patterns of cranial bone growth across ontogeny and possibly predict the mature craniofacial morphology across taxa (Figure 6). This relationship between suture closure and skull morphology becomes increasing complex when multiple suture synostoses are involved. Similarly, abnormal growth of the skull occurs in response to premature fusion of multiple sutures, although growth is stimulated and restricted in a multitude of directions. The skull is instead distorted into a trilobed structure with the overall dysmorphology referred to as the cloverleaf skull or Kleeblattschädel-deformity syndrome (Angle et al., 1967), with the coronal and lambdoid as the most common combination of sutures to fuse (Manjila et al., 2010). It appears evident that the abnormal pattern of suture development and fusion is responsible for encouraging growth in various planes, ultimately warping the skull morphology in a predictable manner based on the location and timing of suture fusion (Figure 6). In humans, not only could suture fusion patterns prove informative in predicting adult cranial morphology, but so too could suture complexity, with a greater complexity in the sagittal suture associated with an altered cranial morphology (lower and broader skull) (Skrzat et al., 2004).

The link between suture closure and overall skull morphology is also highlighted in the field of developmental biology, through the use of mouse mutants where the pattern of suture patency can be disrupted by manipulating transcription factors and signalling pathways (Grova et al., 2012). Alterations to the developmental pathways result in abnormalities in suture fusion timing and drive a number of craniofacial dysmorphologies. A number of mouse models have been established for many of the craniosynostosis syndromes, allowing for an assessment of the interaction between suture fusion and skull morphology (Cornille et al., 2019; Lee et al., 2019). Mutations of the TWIST gene in heterozygous Twist (+/−) mouse mutants, result in premature fusion and an altered overall skull shape reflecting that of Saethre-Chotzen syndrome (el Ghouzzi et al., 1997). More specifically, haploinsufficiency of Twist1, using Twist1 (+/−) mouse mutants, results in coronal synostosis and in turn a widened skull in the left-right lateral direction with a shortening in its anterior-posterior length, similarly reflecting the phenotype of Saethre-Chotzen syndrome (Behr et al., 2011). Given the interaction between Twist and FGF, it is perhaps unsurprising that Ser250Trp substitutions in Fgfr2 using mouse mutant Fgfr2 (250/+) also lead to premature suture fusion (Chen et al., 2003). In the case of the Fgfr2 (250/+) mouse mutant, premature fusion of the coronal suture produces a phenotype mimicking that of Apert syndrome; where a dome-shaped skull morphology and significantly shortened skull in the anterior-posterior axis are observed (Chen et al., 2003). Resultant abnormalities in cranial morphology arising from alterations in suture fusion timings can severely impair craniofacial functioning, as well as creating physiological changes. As in patients with craniosynostosis, the mouse mutants highlight how premature suture fusion can influence the shape of the mature cranial morphology (Figure 6). However, a reduction or to delay in the suture fusion also plays a key role in shaping the skull. In the case of the OPG KO mouse model, an OPG deficiency created a reduced fusion of the interfrontal suture which was coupled with a shortened skull morphology in the anterior-posterior direction (brachycephaly) (Beederman et al., 2015). Evidence from mouse models suggests that sutures, irrespective of the closure status, can serve as targets for developmental changes that affect the overall cranial morphology.

Evidence from pathology and mouse mutants suggest that alterations to suture fusion and morphology have clear implications on the overall cranial morphology. With regard to natural variation, there is much less existing evidence linking the two together. Most papers focussing on sutures to date consider how patterns of suture closure are driven by mechanical and ecological factors across the major clades of animals (Kathe, 1999; Nicolay and Vaders, 2006; Bärmann and Sánchez-Villagra, 2012; Goswami et al., 2013), rather than addressing and considering what consequence suture closure patterns might have on skull shape. For example, in mammals, early fusion of facial sutures in *Pecari* species strengthens the facial region to aid feeding (Bärmann and Sánchez-Villagra, 2012). Such cranial demand and functioning vary across different clades which, in turn, are associated with differing patterns of suture fusion. An interesting example of this adaptive variation can be seen between birds and mammals. Cranial ossification in birds occurs relatively late, after which time the sutures fuse to protect the developed brain. Whereas, in mammals brain growth is extended across many years, in particular in larger mammals, and suture fusion occurs at a later stage in order to support the extended brain maturation period (Morriss-Kay, 2001).

Aside from reports of suture fusion timing involved in supporting cranial functioning, some evidence does exist to support a relationship between suture fusion and morphology, and the overall cranial morphology, across species. In species of *Pecari* (Artiodactyla), suture closure was found to correlate with adult cranial proportions, thus suggesting suture fusion creates a knock-on cranial shape change (Herring, 1974). The early fusing premaxillo-maxillary suture, in peccaries, is associated with a significant increase in palatal length. With the fusion of the middle intermaxillary suture, the palatal width increases in the molar region. This suture fusion and skull growth pattern continues to be the case for other cranial width measurements (distance between supraorbital foramina, anterior nasal width, vault width). Interestingly, this pattern of significant growth subsequent to suture fusion appears to be the case for skull width parameters, rather than skull length parameters, which instead remained largely stable (Herring, 1974). Focussing on mammals, an association between the degree of suture closure level and skull length has been identified within several species of hystricognathous rodents, with suture closure level negatively correlated to cranial length (Wilson and Sánchez-Villagra, 2009). However, it is common for papers to group together the level of suture closure across all suture sites within one specimen, by providing a score for the percentage of average sutural closure per specimen, thus calculating the influence of this average suture closure level on the overall cranial size (Wilson and Sánchez-Villagra, 2009). Grouping the sutures together in this manner to provide an average closure score, is likely to be less informative than considering specific correlations between suture closure and cranial growth patterns. This perhaps explains why the relationship between suture fusion and cranial growth remains so complex with no clear correlation identified (Herring, 1974).

An interesting case study of how suture morphology has helped to shape cranial morphology across evolutionary time, can be observed in Cetacea. Some of the greatest morphological changes in response to the environment observed in any mammalian skull are that of Cetacea. Evolution of telescoping and asymmetry within the cetacean skull have, respectively, facilitated breathing and echolocation enabling cetacean species to thrive within aquatic environments (Miller, 1923; Huggenberger et al., 2017). Telescoping is seen in both odontocetes (toothed whales) and mysticetes (baleen whales), although differs slightly between the clades (Miller, 1923). The phenomenon refers to a shift in the nasal position from the tip of the snout in ancient whales to the top of the head in modern whales. In odontocetes, this involved a posterior displacement and expansion of the premaxilla and maxilla, with extensive overlapping of the maxilla and frontal bones. These extreme changes in cranial morphology are thought to have occurred rapidly during the Oligocene (34–23 Ma) diversification of the modern clades of cetaceans (Churchill et al., 2018; Coombs et al., 2020). During this period of rapid cranial shape change, overlapping at the sutures is thought to have played a central role, which has ultimately altered contact relationships in cetacean cranial bone morphology (Roston and Roth, 2019). In the telescoped areas, sutures are transformed to extreme morphologies; the overlapping bones mean that sutures must span large regions producing an extensive joining surface, also termed “horizontal sutures” (Gatesy et al., 2013). Many questions around telescoping still go unresolved, although it is thought the answers might lie with the sutures, thus stressing the importance of suture morphological changes in craniofacial evolution and development (Roston and Roth, 2019).

It is clear that there is an incredible amount of natural variation across both suture and skull morphology. Even within a single species, changes in cranial morphology can occur across a single year creating seasonal variability in cranial morphology, as reported in the common shrew and least weasels (Pucek, 1963; Dechmann et al., 2017). It is possible suture morphology could also exhibit seasonal fluctuations in-keeping with the cranial adaptations, particularly as sutures are thought to display plasticity to the environment (Roston and Roth, 2019). It has been suggested that cranial morphological changes occurring over evolutionary time could be coupled with alterations in gross suture morphology (Richtsmeier et al., 2006). From a wider evolutionary perspective, finite element analysis (FEA) has shown that sutures have a functional role in responding to and altering strain distribution across lizard skulls (Moazen et al., 2009; Jones et al., 2017). This points toward an ability for sutures to work collectively in adapting to and maintaining a threshold level of strain for bone maintenance across the skull, which in turn may impact the gross suture morphology. Within mammals, there is also a tremendous amount of interspecific variation in both the timing of suture fusion (Wilson and Sánchez-Villagra, 2009; Rager et al., 2014; del Castillo et al., 2015) and suture morphology (Byron, 2009; Buezas et al., 2017). Variation in the timing of suture fusion has been attributed to differing functional pressures and heterochrony (Wilson and Sánchez-Villagra, 2009; Goswami et al., 2013). Given the importance of suture fusion timing in determining craniofacial morphology in pathology and mouse mutants, suture fusion appears to be equally as important as suture morphology in shaping the mammalian skull morphological variation.

Previous work has identified a possible link between craniosynostosis and mammal skull morphological variation (Richtsmeier et al., 2006; Esteve-Altava and Rasskin-Gutman, 2015). As premature suture fusion is hypothesised to be a direct cause of craniosynostosis and, as such, cause modifications to the skull morphology, it is possible that craniosynostosis could act as model for mammal skull evolution (Richtsmeier et al., 2006). Anatomical network models analysing integration and modular organisation of the skull have since shown that craniosynostosis could offer developmental explanations for how changes in suture fusion could occur at a macroevolutionary scale (Esteve-Altava and Rasskin-Gutman, 2015). It has further been suggested that bone loss resulting in higher cranial complexity during development could translate to processes shaping the morphological evolution of the skull (Esteve-Altava et al., 2013). Such losses and bone fusions are thought to impact the skull morphology differently depending on the cranial bones targeted during developmental losses (Esteve-Altava et al., 2014). Within the specific system of mammals, it has been suggested that suture fusion, rather than bone loss, could be the dominant process driving the reduction of cranial bones through the evolutionary history of synapsids (including mammals and their ancestors), a trend referred to as Williston’s law (Gregory et al., 1935; Sidor, 2001; Richtsmeier et al., 2006). This hypothesis requires further testing, although it is clear from multiple sources of evidence, including pathology, mouse mutants and evolutionary trends, that sutures play a key role in the development and evolution of the mature cranial morphology in mammals.

## Possible Mechanisms for Evolutionary Change

The craniofacial variation observed across mammals could arise from evolutionary mechanisms acting on a number of signalling pathways that alter the development and fate of sutures. Multiple signalling pathways could be the target of evolutionary change, such as those which have been identified from multiple mouse models of craniosynostosis (Richtsmeier et al., 2006). During embryonic development, as the initial cranial bones expand and approach each other, it is thought that a gradient of signalling factors is created across the bones, leading to the initial formation of the presumptive sutural matrix (Opperman et al., 1993). However, it is unclear as to which signalling factors are responsible for this formation. Stabilisation of this newly formed presumptive suture is provided by signalling from the dura mater (Opperman et al., 1993). In the absence of a dura mater, sutures continue to form, suggesting that the initiation of suture formation occurs irrespective of dura mater signalling and thus signalling produced at the expanding bone fronts would be necessary for suture formation. This certainly seems to be the case for the sutures formed at anatomical landmarks of the underlying brain tissue (sagittal, interfrontal, and lambdoid). However, in the instance of the coronal suture which forms at the interface of mesoderm and neural crest derived cells, the transcription factor engrailed 1 (En1) is thought to regulate the neural crest and mesodermal cell movements, thus determining the position of the neural crest-mesoderm boundary, which is in turn required for the formation of the coronal suture (Deckelbaum et al., 2012). Moreover, Twist expression during embryonic development also seems to be necessary for the initiation and formation of the coronal suture (Johnson et al., 2000). At the point of appearance of the presumptive suture matrix, a number of growth and transcription factors are known to be present at the suture locale (presumptive suture matrix, underlying dura mater, and approaching bone fronts) including BMP-4, BMP-7, FGF-9 (growth factors), MSX1 and MSX2, and TWIST (transcription factors) (Opperman, 2000). Looking specifically at the Twist pathway, Twist1^+/–^ mouse mutants, produce a reduction in the number of Gli1+ progenitor cells, suggesting there is a crucial link between the TWIST transcription factor and the ability for the suture to act as a site of cranial bone growth (Zhao et al., 2015). As Gli1+ cells are one of the major mesenchymal stem cell populations within the suture mesenchyme that contribute to cranial bone growth, as with the mouse mutants, alterations to the Twist signalling pathway over evolution could have acted to generate craniofacial variation.

Similarly, to suture development, a number of signalling pathways are involved in determining the fate of suture fusion status (patent or fused). Given the significance of these signalling pathways associated with suture fusion status dictating the mature cranial morphology in pathology (craniosynostosis) and mouse models (Grova et al., 2012), such signalling pathways associated with are also likely to be key evolutionary targets for craniofacial morphological change. Sutural-dural interactions have been shown to be required for the maintenance of suture patency throughout postnatal development (Opperman et al., 1993; Roth et al., 1996). Absence of a dura mater instead favours cell proliferation and synthesis of a collagenous extracellular matrix responsible for premature osseous obliteration of the sutures (Opperman et al., 1998). The influence the dura mater has on the maintenance of suture patency and the determination of suture fusion timing is thought to be the result of a number of mediatory signalling factors rather the direct sutural-dural cell interactions (Opperman et al., 1995). Such signalling factors are later dominated by those from the osteogenic fronts (Kim et al., 1998). Variation in the absence or presence of signalling for a number of different factors including TGF-β, FGF, Twist, BMP, noggin, and Wnt collectively determine suture fate. To some extent, fusion of the interfrontal suture is determined by the presence of transforming growth factor (TGF)-β, whereas its absence prevents fusion from occurring (Mehrara et al., 2002). Fibroblast growth factor (FGF) signalling (Fgf1, Fgf2, Fgf3, Fgf4, Fgf9, and Fgf18) is not only necessary for the normal fusion of sutures but is also largely involved in premature suture fusion identified in craniosynostosis syndromes (Carlton et al., 1998; Rice et al., 1998; Greenwald et al., 2001; Lenton et al., 2005). A relationship between FGF and Twist signalling exists, whereby disruption of their interaction can lead to premature suture fusion (Rice et al., 2000). Overexpression of the transcription factor Msx2 enhances osteoblast differentiation thus leading again to premature suture fusion (Liu et al., 1999). Similarly, to TGF-β, bone morphogenetic protein (BMP) favours osteogenesis and thus its presence leads to the osseous obliteration of sutures (Warren et al., 2003). As the activity of BMP is controlled by noggin, whereby noggin activation inhibits BMP signalling, BMP can also be present in patent sutures as well as obliterated sutures. In contrast, the continuous activity of the canonical Wnt pathway is associated with maintaining patent sutures and its inhibition responsible for ossification and suture fusion (Behr et al., 2010, 2011). Differential modulation of this canonical Wnt pathway is thought to be responsible for the different fates of the interfrontal and sagittal sutures in the mouse (Behr et al., 2010). The ultimate fate of sutures is underpinned by the complex interaction of signalling factors released from various sites across the suture locale. Evolutionary interruption of such pathways could result in osseous obliteration of the suture in turn affecting suture function and the potential for cranial bone growth.

The patency and osseous obliteration of a suture is largely controlled by the bone remodelling activity of osteoclasts and osteoblasts. A balance between these bone remodelling cells (osteoclasts and osteoblasts) is required for suture homeostasis and the maintenance of a patent suture (Beederman et al., 2015). Given the role of osteoclasts and osteoblasts in determining suture fusion status in mammals, they have a crucial role in regulating the cranial bone growth capacity at the suture. Many of the signalling pathways discussed above for premature suture fusion, directly impact the balance between osteoblasts and osteoclasts, thus influencing the level of osteogenesis. As the bone forming cells, increased osteoblast activity has often been associated with a premature suture fusion whereby an increase in bone formation is observed at the fused suture (De Pollack et al., 1996). Alterations to a number of signalling factors are known to create perturbations to the bone remodelling process and have since been discovered to lead to changes in suture fusion status. FGF signalling is associated with osteoblast differentiation, if disrupted this is one of the critical pathways known to cause premature suture fusion (Rice et al., 2003). Perturbations in the RANK/RANKL/OPG pathway, which is associated with both osteoblast and osteoclast activity, have more recently been linked to alterations in cranial suture fusion, implying that osteoclasts may also play a critical role at the suture locale (Lee et al., 2011; Beederman et al., 2015). Osteoclast differentiation and activation through the RANK/RANKL interaction maintains suture patency (Lee et al., 2011). Osteoprotegerin (OPG) acts to block this interaction to instead favour osteoblast activity. Therefore, when OPG deficiency occurs, which supports osteoclast differentiation via RANK/RANKL signalling, suture patency is maintained (Beederman et al., 2015). Defects in osteoclast differentiation unsurprisingly are related to premature fusion (Kawata et al., 1998), which have been associated with a downregulation of RANK. In contrast, the presence of RANK appears to be key for the maintenance of suture patency, suggesting a correlation between RANK presence/absence and suture status (Lee et al., 2011). Suture fusion status is therefore unsurprisingly influenced by perturbations in the balance between bone forming osteoblasts and bone resorbing osteoclasts. The extent to which this perturbation impacts skull morphology could be largely dependent upon the timing at which sutural osseous obliteration occurs during ontogeny.

Homeostasis at the suture itself requires a complex interplay between osteoblasts and osteoclasts. We currently have very limited knowledge on this interaction at the suture locale compared to osteoblast and osteoclast involvement in bone homeostasis. Nevertheless, MSCs are thought to have a complex role in mediating the osteoblast-osteoclast interaction within the cranial suture (Guo et al., 2018), given the appropriate signalling MSCs undergo a transition toward an osteoblast fate, a transition that is reliant upon BMP-IHH signalling (Beederman et al., 2013; Guo et al., 2018). It is unsurprising, therefore, that BMP is also found at the fused suture locale given it favours the formation of bone forming cells (osteoblast) (Warren et al., 2003). As the balance between the proliferation of osteoblast precursors and osteogenic differentiation is mediated at the suture, the bone remodelling process has a key involvement in the maintenance of suture patency, enabling the sutures themselves to serve as major signalling centres (Iseki et al., 1997; Zhou et al., 2000). This regulatory function, dictated by suture development and signalling pathways, allows the sutures to function as primary growth centres for the craniofacial bones (Opperman, 2000). Therefore, understanding normal suture development is key to understanding how large scale craniofacial morphological change could occur. Beyond the role of suture fusion, osteoclasts are also thought to be involved in the production of a waveform suture pattern, specifically the osteoclasts that have been identified at the approaching bone fronts (Byron, 2006). As is clear from evolutionary evidence in Cetacea (Roston and Roth, 2019) and from suture morphological diversity across mammals (Byron, 2009; Buezas et al., 2017), suture morphology appears to play a key role in shaping the evolutionary diversity of cranial morphology across mammals. Therefore, it is also possible that this suture morphological variation is controlled on the cellular scale, and thus could be adjusted through the signalling pathways mediating osteoclast activity to produce large scale craniofacial diversity.

It could be hypothesised that the large scale cranial morphological disparity observed across mammals (Goswami, 2006; Koyabu et al., 2014; Usui and Tokita, 2018) is shaped to some degree by the sutural variation, in terms of suture fusion status, developmental processes, and morphology. This variation could result from evolutionary alterations to signalling pathways which underlie suture development and fusion. Such a hypothesis appears to be largely supported by the evidence presented through pathology, mouse mutant models, and natural variation. Nevertheless, much work remains to fully understand the integrated evolution of craniofacial morphology and the adaptive landscape of suture morphology. A greater understanding of this relationship may also aid with future inference of species ecology and life history from suture morphology, fusion, and development and skull form (Figure 5).

## Conclusions

An overwhelming body of research conducted within the fields of development and evolution focusses on the morphology of the skull, including individual skull bones or regions. In contrast, less attention is given to the fibrous sutural joints separating the cranial bones. In light of the ever advancing imaging and geometric morphometric techniques available in the evolutionary developmental biologist’s toolkit, it is becoming increasingly possible to quantitatively and comparatively study sutures to address many of the outstanding questions regarding suture development, morphology, evolution, and importance. Suture development is critical for the normal development of the skull through the provision of interstitial bone growth sites, supporting homeostasis and facilitating repair. Synchronisation in developmental timing between suture formation and cranial bone tissue growth enables a coordinated expansion of the skull and brain. Functionally, sutures support feeding, provide shock absorption during locomotion, and accommodate brain growth. Thus, suture development and skull function are closely intertwined.

In this review, we highlight the importance of sutures, from a number of perspectives, such as morphology, complexity, fusion, and development, in not only supporting the overall functioning of the skull but also in shaping its mature morphology. Evidence from pathology, mutant mouse models, and natural variation, suggests that sutures may act as targets for change in craniofacial morphology, which likely contributes to some of the cranial variation observed across mammals and other vertebrates. While the vast majority of previous work on skull evolution almost entirely overlooks suture morphology and fusion, this review highlights the importance of studying suture morphology and fusion in parallel to skull morphology. As a result, it becomes increasingly important to rethink traditional evolutionary developmental biology questions which consider the skull in isolation. Future work integrating suture development into a comparative evolutionary framework will be instrumental in understanding how developmental mechanisms shaping suture morphology ultimately influences craniofacial form. As critical structures for skull development, function, and morphology, sutures are central to reconstructing the evolution of cranial diversity, but at present, are sorely understudied in evolutionary and developmental biology.

## Author Contributions

HW, AG, and AT designed the outline and aims for the review. HW performed the research necessary for the review and wrote the first draft of the manuscript. AG and AT provided insightful discussion, ideas, and guidance for additional research to produce the final manuscript, as well as contributed to the writing and reading of the final version. All authors approved the final version.

## Conflict of Interest

The authors declare that the research was conducted in the absence of any commercial or financial relationships that could be construed as a potential conflict of interest.
